# Study of microencapsulated fatty acid antimicrobial activity in vitro and its prevention ability of *Clostridium perfringens* induced necrotic enteritis in broiler chicken

**DOI:** 10.1186/s13099-022-00526-9

**Published:** 2023-01-02

**Authors:** Nanshan Qi, Shaobing Liu, Fangquan Yan, Bing Chen, Shilin Wu, Xuhui Lin, Zhuanqiang Yan, Qingfeng Zhou, Shenquan Liao, Juan Li, Minna Lv, Haiming Cai, Junjing Hu, Jianfei Zhang, Youfang Gu, Mingfei Sun

**Affiliations:** 1 Key Laboratory of Livestock Disease Prevention of Guangdong Province, Key Laboratory of Avian Influenza and Other Major Poultry Diseases Prevention and Control, Ministry of Agriculture and Rural Affairs, Institute of Animal Health, Guangdong Academy of Agricultural Sciences, Guangzhou, 510640 Guangdong People’s Republic of China; 2 Guangzhou Wisdom Bio-Technology Co., Ltd, Guangzhou, 510700 Guangdong People’s Republic of China; 3 College of Animal Science and Technology, Anhui Science and Technology University, Fengyang, 233100 Anhui People’s Republic of China; 4 Wen’s Group Academy, Wen’s Foodstuffs Group Co., Ltd., Xinxing, 527400 Guangdong People’s Republic of China

**Keywords:** Necrotic enteritis, *Clostridium perfringens*, Medium chain fatty acid, Antimicrobial activity, Broiler chicken

## Abstract

**Background:**

Necrotic enteritis (NE) is an infectious intestinal disease caused by *Clostridium perfringens* (*C. perfringens*) that is now re-emerging and causing concern within the poultry industry. Previously, the supplementation of antibiotics in feed was the most popular control strategy against *C. perfringens*. However, with the ban on supplementing growth-promoting antibiotics in livestock feed, alternatives to antibiotics will be essential in order to control necrotic enteritis. A possible alternative to antibiotics could be the medium or long chain fatty acids (MCFA or LCFA) as these are able to destroy cell membranes which in turn results in the death of bacteria. In this study, the in vitro antimicrobial activity of different combinations with microencapsulated caprylic acid (C8: 0), capric acid (C10: 0), lauric acid (C12: 0) and myristic acid (C14: 0) against *C. perfringens* and in vivo control the NE-inducing *C. perfringens* in broiler chicken were analyzed.

**Results:**

The minimum inhibitory concentration (MIC) and the minimum bactericidal concentration (MBC) assay results revealed that three different combinations of medium/long chain fatty acids varied in antimicrobial activities against *C. perfringens* type A strain (CVCC52, quality control), *C. perfringens* type A strain (C8-1), *C. perfringens* type G strain (D25) and *C. perfringens* type G strain (MZ1). Specifically, combination of C12: 0 and C14: 0 (C12–14) showed the highest antimicrobial activity against the four strains of *C. perfringens* (MIC ≤ 12.5 μg/mL, MBC = 50 μg/mL), followed by the combination of C10: 0 and C12: 0 (C10–12) (MIC, MBC ≤ 50 μg/mL). The in vivo study, 189 of 818-crossbred chickens that were fed a wheat-based diet and randomly divided into nine groups, with six treatment groups supplemented with either a high dose (1 g/kg) or low dose (0.5 g/kg) of three combinations respectively. The remaining three groups comsisted of a positive group supplement with avilamycin (0.01 g/kg), an infected control and an uninfected control. All chickens were challenged with *C. perfringens* from day 14 to day 17, except those in the uninfected control group. On day 20, the duodenum and jejunum necrotic lesions scores were calculated and the results showed that there was significant decrease in the C12–C14 high dose group (1.43 ± 0.23, 0.48 ± 0.13) and the C10–12 high dose group (1.52 ± 0.19, 0.48 ± 0.11) compared to the infected group (2.86 ± 0.21, 1.20 ± 0.28).

**Conclusions:**

This finding indicated that dietary microencapsulated C12–C14 and C10–C12 could inhibit the growth of *C. perfringens* in chickens, which proves is viability to serve as an alternative to antibiotics used for necrotic enteritis caused by *C. perfringens*.

**Supplementary Information:**

The online version contains supplementary material available at 10.1186/s13099-022-00526-9.

## Introduction

Necrotic enteritis (NE) is one of the most severe intestinal diseases caused by toxin-producing *Clostridium perfringens* (*C. perfringens*) [17]. *C. perfringens* is usually classified into seven toxinotypes (A-G) according to the toxins they produced; including alpha (CPA), beta (CPB), epsilon (ETX), iota (ITX), enterotoxin (CPE) and NetB. *C. perfringens* type A (CPA producing type), C (CPA and CPB producing type) and G (CPA and NetB producing type) are considered the main causative toxinotypes of NE in poultry production [7], Kiu and Hall [8]). *C. perfringens* can damage the intestinal surface and induce necrotic lesions, resulting in reduced growth rate, impaired feed absorption and increases in broilers mortality of more than 50%, causing huge losses for the poultry industry of approximately six billion US dollars annually [1, 4].

Additive of antibacterial growth promoter (AGP) such as enramycin, zinc bacitracin and virginiamycin in feeds have been an important strategy for the control of NE in poultry for decades [16]. However, with the overuse of AGPs, an increasing number of problems have occurred, including the development and expansion of drug resistance and drug residue which ultimately resulted in the ban of AGP use in food animal production in numerous countries in order to protect human health [4]. Due to the restriction of AGPs, the poultry industry faces an ever growing need to find a AGP alternative as NE is becoming a resurgent infectious disease which causes significant increase in morbidity and mortality, reflecting huge financial losses for the global poultry industry year after year [1]. Therefore, an alternative to antibiotics to control NE has become increasingly urgently needed.

Medium chain fatty acids (MCFA) including C6, C8, C10, C12 and C14 (myristic acid) are naturally found in many plants oil or dairy products [14]. MCFA and myristic acid can react with GPR84 to influence the immune function of a host cell, which plays an important role in stimulating cell-mediated immunity, as well as modulating the intestinal microbiota to improve the host’s immunity [12]. The hydrophilic and hydrophobic characteristics of both MCFA and myristic acid play a key role in its antimicrobial activity, as when it is inserted into the membrane bi-layer of bacteria, it leads to lysis of the cell and thus cell death [2]. Microencapsulation is an important technology widely used for the lipid delivery system, which prevents the oxidation of fatty acids and maintains the quality of lipid after extraction from oil seed and processing. However, what is less known is the antimicrobial activity of microencapsulated MCFA and myristic acid against *C. perfringens*.

Therefore, in this study, the in vitro antimicrobial activity of different combinations of microencapsulated caprylic acid (C8: 0), capric acid (C10: 0), lauric acid (C12: 0) and myristic acid (C14: 0) against *C. perfringens* and in vivo control of the *C. perfringens* induced NE in broiler chicken were analyzed. This current study aimed to find out a combination of MCFA and myristic acid which hopefully will serve as an ideal alternative for antibiotics against *C. perfringens*-induced necrotic enteritis in the poultry industry.

## Results

### MIC and MBC results of microencapsulated products against *C. perfringens *in vitro

The antimicrobial activity of microencapsulated products (C8–C12–C14, C12–C14 and C10–C12) and avilamycin against four strains of *C. perfringens*: CP-D25, CP-MZ1, CP-C8-1 and CP-CVCC52 (quality control) in vitro were tested using MIC and MBC. As shown in Table 1, the MIC value of C8–C12–C14 against CP-D25, CP-MZ1, CP-C8-1 and CP-CVCC52 were 0.1 mg/mL, 0.1 mg/mL, 0.5 mg/mL and 0.2 mg/mL respectively, and the MBC values were 0.1 mg/mL, 0.2 mg/mL, 1.0 mg/mL and 0.2 mg/mL respectively. The MIC values of C12-C14 against CP-D25, CP-MZ1, CP-C8-1 and CP-CVCC52 were 0.00625 mg/mL, 0.0125 mg/mL, 0.00625 mg/mL and 0.003125 mg/mL respectively, and the MBC values were 0.05 mg/mL in all groups. The MIC value of C10-C12 against CP-D25, CP-MZ1, CP-C8-1 and CP-CVCC52 were 0.05 mg/mL, 0.05 mg/mL, 0.5 mg/mL and 0.025 mg/mL respectively, while the MBC values were 0.1 mg/mL, 0.1 mg/mL, 0.5 mg/mL and 0.1 mg/mL respectively. In addition, the MIC value of avilamycin against CP-D25 and CP-CVCC52 were 0.00625 mg/mL, and the MBC values were 0.05 mg/mL, however, CP-MZ1 and CP-C8-1 were found to resistant to avilamycin.

**Table 1:** MICs and MBCs of different treatments against different strains of *C. perfringens*

Treatment	CP-D25^**#**^		CP-MZ1^**#**^		CP-C8-1^**#**^		CP-CVCC52^**#**^	
	MIC^**#**^ (mg/mL)	MBC^**#**^ (mg/mL)	MIC^**#**^ (mg/mL)	MBC^**#**^ (mg/mL)	MIC^**#**^ (mg/mL)	MBC^**#**^ (mg/mL)	MIC^**#**^ (mg/mL)	MBC^**#**^ (mg/mL)
C8-C12-C14	0.1	0.1	0.1	0.2	0.5	1.0	0.2	0.2
C12-C14	0.00625	0.05	0.0125	0.05	0.00625	0.05	0.003125	0.05
C10-C12	0.05	0.1	0.05	0.1	0.5	0.5	0.025	0.1
Avilamycin	0.00625	0.05	Resistant	Resistant	Resistant	Resistant	0.00625	0.05

*MIC* minimum inhibitory concentration, *MBC* minimum bactericidal concentration

^#^CP-D25, *C. perfringens* type G strains, sensitive to Avilamycin. CP-MZ1, *C. perfringens* type G strains, resistance to Avilamycin. CP-C8-1, *C. perfringens* type A strains, resistance to Avilamycin. CP-CVCC52, *C. perfringens* type A strains, sensitive to Avilamycin

### Effect of microencapsulated products on growth performance in broiler chickens

**Fig. 1:**
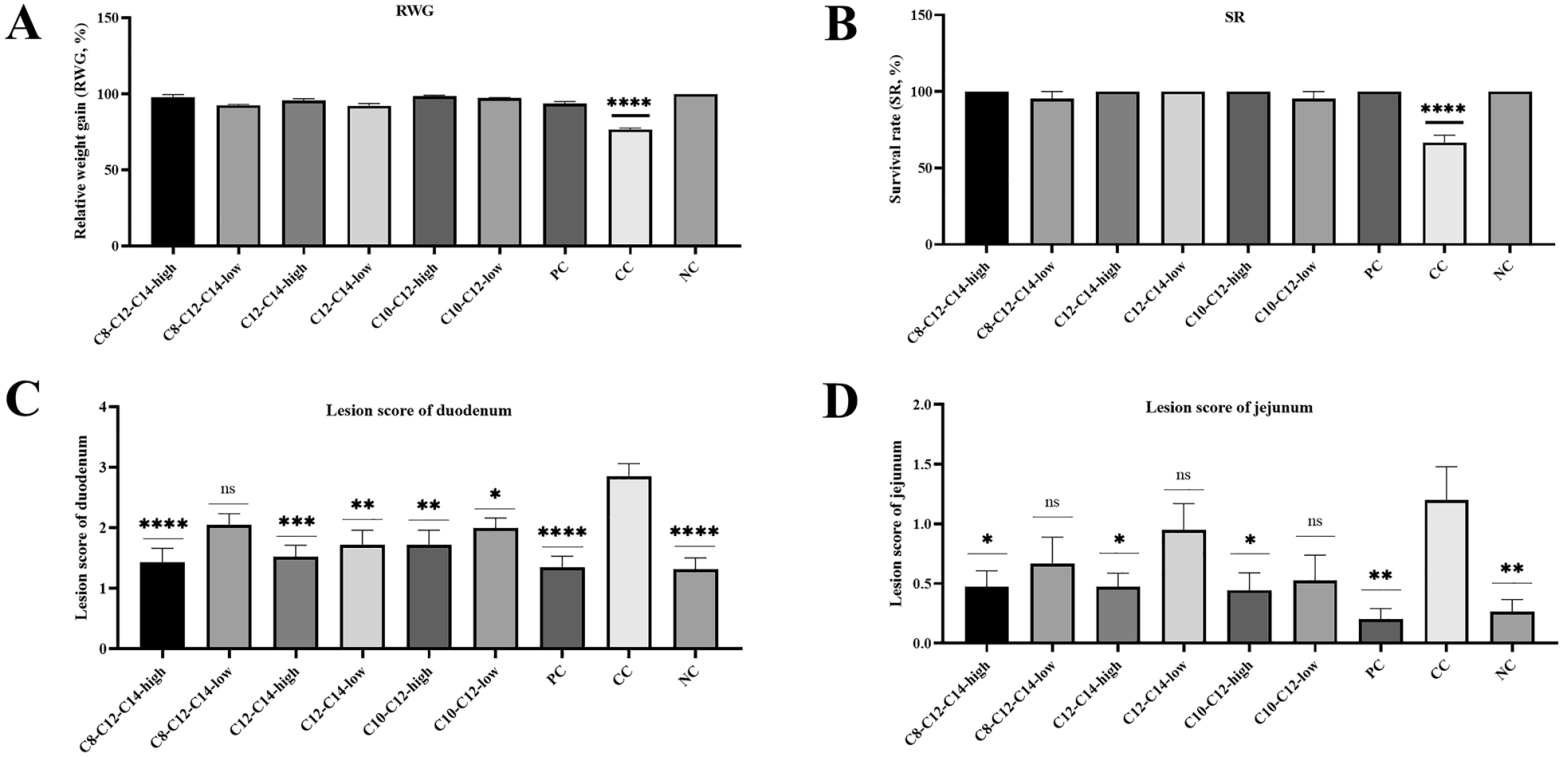
Comparative values of the relative weight gain (RWG), survival rate (SR), lesion score of duodenum and jejunum. **A** relative weight gain (RWG) in birds of different treatments. **B** survival rate (SR) in birds of different treatments. **C** lesion score of duodenums in birds of different treatments. **D** lesion score of jejunums in birds of different treatments. *PC* avilamycin-positive control. *CC* challenge control. *NC* no additive supplementation and no challenge. Astricts signify statistical significance in comparison to the control group (*p < 0.05, **p < 0.005, ***p < 0.0005, ****p < 0.0001)

The results of the relative weight gain (RWG) and survival rate (SR) are shown in Fig. 1A and B. There was no significant difference in SR and RWG among all treatment groups compared with the negative control (no additive supplementation and no challenge) group. The challenge group (CC) with no treatment showed a significant decrease in the SR (66.67 ± 4.76%, *p* < 0.0001) and RWG (76.56 ± 1.03%, *p* < 0.0001) compared to the negative control group (100.00% ± 0.00), which is shown in Table 2.

**Table 2:** Comparative values of the survival rate (SR), relative weight gain (RWG), lesion score of duodenum and jejunum

Groups	SR^#^ (%)	RWG^#^ (%)	Lesion score	
			Duodenum	Jejunum
C8-C12-C14-high	100.00 ± 0.00	97.92 ± 1.73	1.43 ± 0.23^****^	0.48 ± 0.13^*^
C8-C12-C14-low	95.24 ± 4.76	92.65 ± 0.58	2.05 ± 0.18^ ns^	0.67 ± 0.22^ ns^
C12-C14-high	100.00 ± 0.00	95.79 ± 1.16	1.52 ± 0.19^***^	0.48 ± 0.11^*^
C12-C14-low	100.00 ± 0.00	92.27 ± 1.53	1.72 ± 0.24^**^	0.95 ± 0.22 ns
C10-C12-high	100.00 ± 0.00	98.50 ± 0.76	1.72 ± 0.24^**^	0.44 ± 0.15^*^
C10-C12-low	95.24 ± 4.76	97.17 ± 0.48	2.00 ± 0.16^*^	0.52 ± 0.21^ ns^
PC^**#**^	100.00 ± 0.00	93.69 ± 1.25	1.35 ± 0.18^****^	0.2 ± 0.09^**^
CC^**#**^	66.67 ± 4.76^*^	76.56 ± 1.03^*^	2.86 ± 0.21	1.20 ± 0.28
NC^**#**^	100.00 ± 0.00	100.00 ± 0.00	1.32 ± 0.19^****^	0.26 ± 0.10^**^

^#^*SR* survival rate, *RWG* relative weight gain, *PC* avilamycin-positive control, *CC* challenge control, *NC* no additive supplementation and no challenge

The results were reported as the mean ± SEM, astricts signify statistical significance in comparison to the control group (*p < 0.05, **p < 0.005, ***p < 0.0005, ****p < 0.0001)

### Effect of microencapsulated products on gross lesions in broiler chickens

The results of gross lesions of duodenum and jejunum are shown in Fig. 1C and D. The NE lesion scores of duodenum and jejunum in the high dose of C8–C12–C14 (1.43 ± 0.23, *p* < 0.0001; 0.48 ± 0.13, *p* < 0.05), C12–C14 (1.52 ± 0.19, *p* < 0.0005; 0.48 ± 0.11, *p* < 0.05), C10–C12 (1.72 ± 0.24, *p* < 0.005; 0.44 ± 0.15, *p* < 0.05), Avilamycin-positive control (1.35 ± 0.18, *p* < 0.0001; 0.2 ± 0.09, *p* < 0.005), and NC (1.32 ± 0.19, *p* < 0.0001; 0.26 ± 0.10, *p* < 0.005) groups were significantly lower compared to the CC group (2.86 ± 0.21; 1.20 ± 0.28). No significant difference was observed in the lesion scores of jejunums in low dose of C8–C12–C14 (0.67 ± 0.22, *p* > 0.05), C12–C14 (0.95 ± 0.22, *p* > 0.05) and C10–C12 (0.52 ± 0.21, *p* > 0.05) groups, which is shown in Table 2.

## Discussion

MCFA and myristic acid are both derived from plant and animal natural products, they contain antibacterial activities, which are proven to be beneficial for the intestinal health of animals. Food safety is of global concern and it is strongly advocated that industries reduce/replace antibiotics consumption for animals. MCFA and myristic acid are promising alternatives for the protection of intestinal health in modern livestock [6]. However, there are few studies outlining the benefits of MCFA and myristic acid or microencapsulated fatty acids on the prevention and control of chicken necrotic enteritis. To find a novel strategy to control the chicken necrotic enteritis, this study detected the antibacterial activity of different kinds of microencapsulated fatty acids against *C. perfringens *in vitro and control *C. perfringens* induced NE in broiler chicken.

*Clostridium perfringens* is an opportunistic pathogen that can survive and proliferate in the cecum of healthy animals. It has been shown that toxin-type A and G *C. perfringens* are the most important toxin types that endanger the development of chickens [5], Lee and Lillehoj [9]). Therefore, both type A and G of *C. perfringens* wild isolated strains are important models for screening potential antibacterial products. In the present study, the results of antibacterial activity of MCFA and myristic acid showed that microencapsulated C8–C12–C14, C12–C14 or C10–C12 had efficient bacteriostatic and bactericidal effects on the two toxin-types of *C. perfringens* from different isolates. This finding provides the fundamental support required for further screenings of anti-Clostridial preparations for clinical application.

In addition, supplementation of antibiotics has been the chosen method to prevent and control Clostridium for decades, which has led to the emergence of a large number of antibiotic-resistant strains of *C. perfringens* in the field [3, 11, 15]. Therefore, isolation of antibiotic-resistant strains are important for screening effective antibacterial products. In this study, the antibacterial activity of MCFA and myristic acid against *C. perfringens* CP-MZ1 and CP-C8-1 which are resistant to avilamycin were tested using MIC and MBC in vitro, while *C. perfringens* CP-CVCC52 and CP-D25 which are sensitive to avilamycin were used as control bacteria. The results showed that microencapsulated C8–C12–C14, C12–C14 or C10–C12 displayed efficient antibacterial effects on the wild isolated avilamycin-resistant strains, suggesting that microencapsulated combinations of different MCFA and myristic acid have the potential for clinical application as a green antibacterial to replace antibiotics such as avilamycin.

MIC and MBC in vitro are high-throughput and broad-spectrum strategies for antibacterial screening, however, they may not be as effective as in vivo animal trial. In our previous study, we screened some highly efficient bactericidal plant essential oils and short-chain fatty acids in vitro, however they did not show effective protection on *C. perfringens* induced necrotic enteritis when managing in vivo experiments (unpublished). In this study, we screened C8: 0, C10: 0, C12: 0 and C14: 0 as effective antibacterial agents through in vitro MIC and MBC tests and found that they could also function in the challenged animal model. However, C10–C12 did not show a significant difference in the protection of gut health in vivo, although it showed the lowest dose of antibacterial activity against *C. perfringens* using MIC and MBC tests in vitro. Therefore, these results suggested that the combination of in vitro high-throughput screening and in vivo challenged animal model is of great significance for high effective antibacterial drugs screening and enhance the reliability.

## Conclusions

Medium chain fatty acids (MCFA) and myristic acid from natural sources can destroy cell membranes resulting in the death of bacteria, this presents the possibility of being a suitable alternative to antibiotics. The results of this study indicate that microencapsulated combination with different kinds of MCFA and myristic acid could effectively inhibit the wild isolated avilamycin-resistant strains of *C. perfringens* type A and G in vitro and it could also effectively protect the chickens against the *C. perfringens* challenge in vivo. Therefore, with the ban on supplementing growth-promoting antibiotics in livestock feed regulation, combination with MCFA and myristic acid promising to become an alternative to antibiotics for controlling necrotic enteritis in the poultry industry.

## Materials and methods

### Bacterial strains and the growth conditions

Three strains of *C. perfringens*, CP-D25, CP-MZ1 and CP-C8-1 were isolated from the intestinal tract of chicken with necrotic enteritis in southern China, in which CP-D25, CP-MZ1 were identified as toxin type G, and CP-C8-1 was identified as toxin type A. The quality control strain *C. perfringens* type A CP-CVCC52 was purchased from China Veterinary Culture Collection Center, China Institute of Veterinary Drug Control, Beijing, China. All strains were deposited in the Parasitology Laboratory of Institute of Animal Health, Guangdong Academy of Agricultural Sciences.

All strains were grown in Tryptose Sulfite Cycloserine (TSC, Guangdong Huankai Microbial Sci. and Tech. Co.,Ltd.) agar solid medium and Fluid Thioglycollate medium (FT, Guangdong Huankai Microbial Sci. and Tech. Co., Ltd.) at 37 ℃ in anaerobic conditions.

### Medium chain fatty acids MCFA, myristic acid and antibiotic

For fatty acids microencapsulation, Tween 80 was used as a surfactant, and colloidal silicon was employed as an anti-caking agent. The three microencapsulated products of different combination with MCFA or myristic acid (from GuangZhou Wisdom Bio-Technology Co., Ltd., Guangzhou, China) are: Wisdem Modified MCFA-1 (C8-C12-C14, 40 mg/mL), Wisdem Modified MCFA-2 (C12–C14, 8 mg/mL), and Wisdem Modified MCFA-3 (C10–C12, 20 mg/mL). Maxus (Avilamycin, 10%) were purchased from Elanco Animal Health Incorporated.

### The in vitro antimicrobial effect of the microencapsulated products

To detect the anti-clostridial activity of microencapsulated products or avilamycin in vitro, the minimum inhibitory concentration (MIC) and minimum bactericidal concentration (MBC) against *C. perfringens* type A and G (CP-CVCC52, CP-C8-1, CP-D25 and CP-MZ1) was measured in triplicate using broth microdilution as described by Radaelli et al. with some modifications [13]. *C. perfringens* were grown overnight at 37 ℃ anaerobically on the TSC plates, to which a single colony of the *C. perfringens* was then inoculated from the TSC plate and cultured overnight anaerobically in FT broth. For the MIC assay, a twofold serial dilution of microencapsulated products and avilamycin was performed using FT broth to achieve a final working concentration ranging from 4 to 0.039 mg/mL of C8–C12–C14, 0.8 mg/mL to 0.0078 mg/mL of C12–C14 and avilamycin, and 2 mg/mL to 0.0195 mg/mL of C10–C12, 100 μL each well. Subsequently, 100 μL of the *C. perfringens* suspension broth (1 × 10^7^ CFU/mL) was added to each well of 96-well microplates with mixing to achieve a final concentration of 5 × 10^5^ CFU/mL incubated at 37 ℃ for 24 h under anaerobic conditions. After incubation, the optical density of 600 nm wavelength of each well was measured. MIC values were defined as the lowest concentration of the microencapsulated products that inhibited visible bacterial growth after 24 h incubation.

Based on the MIC results, 100 μL cultures of the minimum inhibition concentration group and five of its higher concentrations were plating on TSC agar and incubated anaerobically for 24 h. MBC values were determined by the colony counts on TSC agar plates, in which the corresponding MIC with less than 4 colonies is considered as the MBC of the microencapsulated products [13].

## The effect of microencapsulated products in control of *C. perfringens* induced NE in broiler chicken

### Animals and experimental design

A total of 189 818-crossbred chickens at the age of 1 day were obtained from a commercial hatchery named Wen's Foodstuffs Group Co., Ltd. The birds were randomly divided into 9 groups consisting of 3 replicates with 7 birds per replicate, and kept in separate steel cages. They were fed a wheat-based diet (Additional file 1: Table S1), with soybean meal as the protein source and their diet was excluded from additives, such as antibiotics and/ or anticoccidials. The microencapsulated products or Avilamycin were mixed with feed from day eight according to the experimental design shown in Table 3.

**Table 3:** Trial designs

Groups	Number of chickens	Treatment dose	Treatment day	*C. perfringens* challenge	Sampling day
C8-C12-C14-high	21	1 g/kg feed	8–20	Day 14–17	20
C8-C12-C14-low	21	0.5 g/kg feed	8–20	Day 14–17	20
C12-C14-high	21	1 g/kg feed	8–20	Day 14–17	20
C12-C14-low	21	0.5 g/kg feed	8–20	Day 14–17	20
C10-C12-high	21	0.5 g/kg feed	8–20	Day 14–17	20
C10-C12-low	21	0.3 g/kg feed	8–20	Day 14–17	20
PC^**#**^	21	10 g/kg feed	8–20	Day 14–17	20
CC^**#**^	21	–	–	Day 14–17	20
NC^**#**^	21	–	–	–	20

^**#**^*PC* avilamycin-positive control, *CC* challenge control, *NC* no additive supplementation and no challenge

### *C. perfringens* challenge

The challenge trial was carried out using NetB positive *C. perfringens* G25 strain as described by Mudassar et al. with some modifications [10]. All birds in each group, except the negative control group, were orally given 3 mL (2.5 × 10^8^ CFU/mL) of freshly prepared culture starting from day 14 for 4 consecutive days. Birds were given 3 mL FT broth orally in the negative control group.

### Survival rate and growth performance assessment

The bird’s performance was evaluated by calculating the SR and RWG for each group. The birds were weighed on day one and on the day of slaughter (day 20). SR (%) of each group = (The numbers of birds at the end/ the numbers of birds on day one) × 100%, RWG (%) = (weight gain in each treated group/ weight gain in negative control group) × 100%.

### Lesion scoring

On day 20, birds from each group were euthanized by CO_2_ in order to evaluate the NE lesions, and the gross intestinal lesion score (duodenum, jejunum and ileum). The lesion scores ranged from 0 (no lesions grossly), 1 (congestion of intestinal mucosa), 2 (1 to 5 foci), 3 (6 to 15 foci) and 4 (16 or more foci) as described by Mohiuddin et al. [10].

### Statistical analysis

The differences among groups in bird performance and lesion score were calculated using One-way ANOVA with GraphPad Prism version 9 (GraphPad Software Inc., San Diego, CA, USA). The results were reported as the mean ± SEM, asterisks signify statistical significance in comparison to the control group (*p < 0.05, **p < 0.005, ***p < 0.0005, ****p < 0.0001).

## Supplementary Information

**Additional file 1: Table S1.** Ingredients and composition of wheat-based diet

## Data Availability

All relevant data were included in the paper.
