# Supplementary material for: Study of microencapsulated fatty acid antimicrobial activity in vitro and its prevention ability of Clostridium perfringens induced necrotic enteritis in broiler chicken
Source: Gut Pathog. 2023 Jan 2;15:1. doi: 10.1186/s13099-022-00526-9 (PMC9808942; doi:10.1186/s13099-022-00526-9)
Supplement: Supplementary file 1 — Additional file 1: Table S1. Ingredients and composition of wheat-based diet [file 13099_2022_526_MOESM1_ESM.docx]

**Table S1. Ingredients and composition of wheat-based diet**

| **Ingredients** | | **(%)** | |
| --- | --- | --- | --- |
| Wheat | | 601.09 | |
| Soybean meal (46%) | | 327.30 | |
| Limestone | | 16.06 | |
| Soy-oil | | 33.30 | |
| Monodicalcium phosphate | | 6.20 | |
| L-Lysine Sulphate (70%) | | 4.20 | |
| Premix^1^ | | 4.00 | |
| Salt | | 1.80 | |
| DL -Methionine (98%) | | 2.35 | |
| Choline-Cl (60%) | | 0.80 | |
| Sodium bicarbonate | | 1.50 | |
| Threonine (98%) | | 1.00 | |
| Anti-mildew | | 0.40 | |
| **SUM** | | **1000.00** | |
| **Nutrients** | **Unit** | | **Value** |
| Dry matter | % | | 88.90 |
| Crude protein | % | | 18.00 |
| Crude fat | % | | 6.12 |
| Crude fiber | % | | 2.64 |
| Ca | % | | 0.85 |
| P (total) | % | | 0.49 |
| NPP | % | | 0.260 |
| Na | % | | 0.16 |
| K | % | | 0.72 |
| Electrolytes | mEq/kg | | 182 |
| ME | kcal/kg | | 3050 |
| Lysine | % | | 1.09 |
| DLys-P | % | | 0.97 |
| D(M+C)-P | % | | 0.76 |
| DThr-P | % | | 0.63 |
| DArg-P | % | | 0.97 |
| DTrp-P | % | | 0.18 |
| DIle-P | % | | 0.67 |
| DVal-P | % | | 0.73 |
| DLys/CP-P |  | | 0.054 |
| DMC/DLy-P |  | | 0.781 |
| DThr/DLy-P |  | | 0.650 |
| DArg/DLy-P |  | | 1.001 |
| DTrp/DLy-P |  | | 0.181 |
| DIle/DLy-P |  | | 0.688 |
| DVal/DLy-P |  | | 0.755 |

^1^Provided per kg of premix: vitamin A, 16,000 IU; vitamin D3, 3,200 IU; vitamin E, 35IU; vitamin K, 5 mg; calcium pantothenic acid, 16 mg; riboflavin, 6 mg; niacin, 32 mg; vitamin B12, 20 μg; biotin, 128μg; Cu, 288 mg; Fe, 281mg; Mn, 49 mg; Se, 0.3 mg and I, 0.3 mg.
